# John C. Storey, M.D., D.D.S.

**Published:** 1897-05

**Authors:** 


### JOHN C. STOREY, M.D., D.D.S.

The profession of dentistry has had to mourn the loss of many of its devoted adherents, especially during the past two or three years, but it is thought few will be missed more than John C. Storey in his own special field of work.

His reputation had long since passed the bounds of his State, and his name had become a familiar sound wherever dentistry was understood and appreciated.

Those who were brought in personal contact with him could not fail to be impressed with his energy, intellectual force, and genial companionship.

His professional attainments were a power for good in his own State, ever tending towards a higher standard of culture and a broader professional perception.

Our fellow-workers and companions are rapidly joining the host of the immortals, but their transition, while it leaves its shadow, teaches us the lesson that he lives best in the memory of his colleagues whose work has continued up to the hour and to the demand, "Come up higher!" Of such was John C. Storey.

